# Host phylogeny, habitat, and diet are main drivers of the cephalopod and mollusk gut microbiome

**DOI:** 10.1186/s42523-022-00184-x

**Published:** 2022-05-08

**Authors:** Woorim Kang, Pil Soo Kim, Euon Jung Tak, Hojun Sung, Na-Ri Shin, Dong-Wook Hyun, Tae Woong Whon, Hyun Sik Kim, June-Young Lee, Ji-Hyun Yun, Mi-Ja Jung, Jin-Woo Bae

**Affiliations:** Department of Life and Nanopharmaceutical Sciences and Department of Biology, Kyung Hee University, Seoul, 130-701 Korea

**Keywords:** Cephalopod, Gut microbiota, Mollusca, Phylosymbiosis, *Photobacterium*, *Mycoplasma*

## Abstract

**Background:**

Invertebrates are a very attractive subject for studying host-microbe interactions because of their simple gut microbial community and host diversity. Studying the composition of invertebrate gut microbiota and the determining factors is essential for understanding their symbiotic mechanism. Cephalopods are invertebrates that have similar biological properties to vertebrates such as closed circulation system, an advanced nervous system, and a well-differentiated digestive system. However, it is not currently known whether their microbiomes have more in common with vertebrates or invertebrates. This study reports on the microbial composition of six cephalopod species and compares them with other mollusk and marine fish microbiomes to investigate the factors that shape the gut microbiota.

**Results:**

Each cephalopod gut consisted of a distinct consortium of microbes, with *Photobacterium* and *Mycoplasma* identified as core taxa. The gut microbial composition of cephalopod reflected their host phylogeny, the importance of which was supported by a detailed oligotype-level analysis of operational taxonomic units assigned to *Photobacterium* and *Mycoplasma*. *Photobacterium* typically inhabited multiple hosts, whereas *Mycoplasma* tended to show host-specific colonization. Furthermore, we showed that class *Cephalopoda* has a distinct gut microbial community from those of other mollusk groups or marine fish. We also showed that the gut microbiota of phylum *Mollusca* was determined by host phylogeny, habitat, and diet.

**Conclusion:**

We have provided the first comparative analysis of cephalopod and mollusk gut microbial communities. The gut microbial community of cephalopods is composed of distinctive microbes and is strongly associated with their phylogeny. The *Photobacterium* and *Mycoplasma* genera are core taxa within the cephalopod gut microbiota. Collectively, our findings provide evidence that cephalopod and mollusk gut microbiomes reflect host phylogeny, habitat, and diet. It is hoped that these data can contribute to future studies on invertebrate–microbe interactions.

**Supplementary Information:**

The online version contains supplementary material available at 10.1186/s42523-022-00184-x.

## Background

Host and bacteria have coexisted for a long time and have evolved together. Since microbiota play an important role in immune response [1] and metabolic regulation [2] within host organisms, it is essential that research is conducted on factors that can affect the gut microbiota. In vertebrates, the gut microbiota composition is influenced by host diet [3], lifestyle [4], habitat [5], and genetic factors [6]. However, little is known about the microbiomes of invertebrates, which account for 90% of all known animal species. Additionally, most studies on invertebrate microbiomes mainly focus on model organisms such as *Drosophila spp.* [7, 8].

In general, invertebrate microbial communities are relatively simple [9–11]. Although invertebrates are frequently exposed to an abundance of microbes within their habitats, very few bacterial species are found within their digestive tracts. Given that there is no difference in the number of microbial species present on the surface of vertebrates and invertebrates, it is clear that their simple composition of gut microbiota is due to symbiotic bacteria selection by the host [12]. Therefore, interactions between the host and gut bacteria and their mechanisms can be more readily elucidated in invertebrates. Furthermore, invertebrates provide numerous study opportunities for researchers because of their sheer abundance and diversity [13].

Since invertebrates live in almost every environment, there are an extraordinary number of host-microbial symbiosis cases that have evolved so that the host organisms can adapt to specific environments [14]. Studying the cases of various host-microbial symbiosis in invertebrates will provide a much better understanding of the various mechanisms by which microbes are involved in host in host development [15], adaptation [16] and even survival [17]. Conducting research on the composition of invertebrate gut microbiota and their determining factors is a prerequisite for understanding their symbiotic mechanisms.

In this study, we characterized the microbiomes of cuttlefish (*Sepia esculenta*, order *Sepiida*), the beka squid (*Loliolus beka*, order *Teuthida*), the inshore squid (*Uroteuthis edulis*, order *Teuthida*), the Japanese flying squid (*Todarodes pacificus*, order *Teuthida*), the common octopus (*Octopus vulgaris*, order *Octopoda*), and the whiparm octopus (*Octopus variabilis*, order *Octopoda*). We aimed to investigate whether host phylogeny is reflected in their microbiome by comparing whether cephalopods belonging to the same species or order have similar microbial communities. Since all members of class *Cephalopoda* known to date are carnivorous and live in marine environments, we obtained other mollusk microbiome data from previous studies. Microbiome data of the bone-eating snail (marine carnivore), emerald sea slug (marine herbivore, class *Gastropoda*), freshwater snail (freshwater herbivore/omnivore, class *Gastropoda*), Hawaiian land snail (terrestrial herbivore, class *Gastropoda*), oyster (marine omnivore, class *Bivalvia*) and fish (marine vertebrates with varied diets) were downloaded and compared with our cephalopod data to evaluate the influence of host phylogeny (inter-class level) living environment and diet on microbiome composition.

Cephalopods are interesting study targets because they are the only group within the *Mollusca* phylum with a closed circulation system [18], an advanced nervous system [19], and a well-differentiated digestive system [20], characteristics that have more in common with vertebrates. We also investigated whether the microbiomes of cephalopods have more in common with invertebrates or vertebrates.

## Results

### Characteristics of the cephalopod gut microbiota

After sequence quality-filtering (and excluding sequences that were found fewer than 15 times in the entire sample), a total of 3,661,327 high-quality reads from 30 samples (6 samples per cephalopod species) were generated, with a mean sample depth of 122,044 and a standard deviation of 20,693.

After rarefaction, 76,381 high-quality sequences were clustered into 1,835 operational taxonomic units (OTUs) at a 97% sequence-identity threshold (357 ± 103 OTUs per sample). Faith’s phylogenetic diversity index (PD), an alpha diversity measure, was used to estimate bacterial species richness (Additional file 1: Fig. S1). The Chao1 metric reached a plateau after 75,000 reads, suggesting that the depth of coverage was sufficient for capturing nearly all the biological diversity within samples (Additional File 1: Fig. S2).

**Fig. 1:**
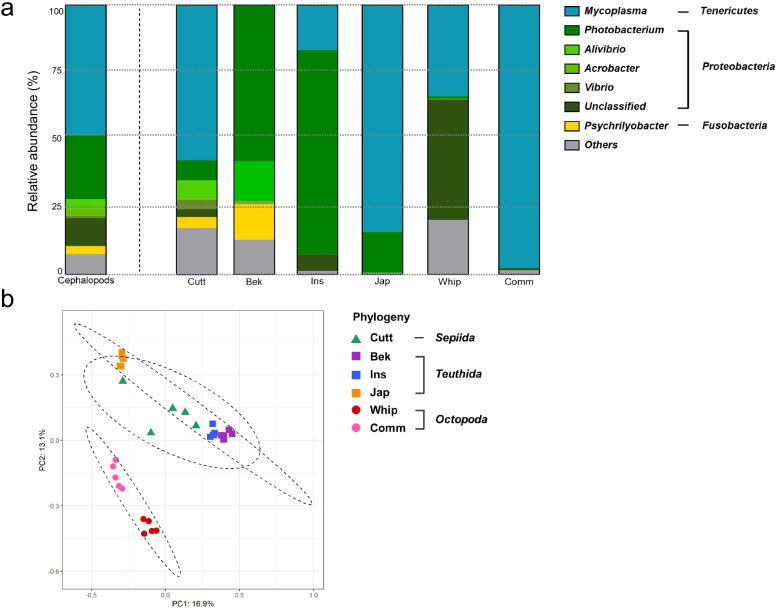
Gut microbial community structure of cephalopods. **a** Bar charts of the relative abundance of bacterial genera in six cephalopod species and the overall gut microbial composition of cephalopods. Only genera with a relative abundance of > 0.5% are shown; those with < 0.5% are classified as “Others.” **b** Principal coordinates analysis (PCoA) of binary Jaccard distances between cephalopod samples. The colored dots and ellipses in PCoA represent the host cephalopod species and their orders. Abbreviations: Cutt, cuttlefish; Bek, beka squid; Ins, Inshore squid; Jap, Japanese flying squid; Whip, whiparm squid; Comm, common octopus

Cuttlefish and beka squid showed higher gut bacterial diversity than those of other cephalopod species, while the Japanese flying squid showed the lowest bacterial diversity. The whiparm octopus and common octopus, members of the order *Octopoda*, had similar diversity levels. Overall, *Tenericutes* (50.0 ± 7.0% relative abundance) and *Proteobacteria* (43.2 ± 6.5%) were the phyla found most frequently in samples (Additional File 1: Fig. S3), while *Mycoplasma* (50.0 ± 7.0%) and *Photobacterium* (23.8 ± 6.4%) were the most common genera. However, the predominant bacteria in the gut microbial communities varied depending on the cephalopod host species (Fig. 1a, Additional File 1: Fig. S4). For example, cuttlefish microbiota was dominated by *Mycoplasma* (*Tenericutes*, 57.4 ± 13.5%); beka squid contained *Photobacterium* (*Proteobacteria*, 58 ± 16.5%), *Aliivibrio* (*Proteobacteria*, 14.7 ± 11.2%), and *Psychrilyobacter* (*Fusobacteria*, 13.2 ± 6.9%), while inshore squid contained *Photobacterium* (*Proteobacteria*, 75.9 ± 7.1%) and *Mycoplasma* (*Tenericutes*, 16.6 ± 3.5%). The *Mycoplasma* (*Tenericutes*, 84.2 ± 8.8%) and *Arcobacter* (*Proteobacteria*, 14.8 ± 8.7%) were found in abundance in the Japanese flying squid., while in the whiparm and common octopus, *Mycoplasma* (*Tenericutes*, 43.7 ± 7.2% and 97.5 ± 0.8%, respectively) were most abundant.

### Cephalopod gut microbial communities reflect host phylogeny

The taxonomic profile clearly shows that although each cephalopod species have a unique microbial community, they all share a core bacteria (Fig. 1a), an observation supported by our beta-diversity analysis. The cephalopod gut microbial communities were clustered according to host species in a principal coordinates analysis (PCoA) of binary Jaccard distances (Fig. 1b). Additionally, samples belonging to the same host order were plotted close to each other. Accordingly, our PCoA analysis suggested that the microbial composition of cephalopod species would be determined by host phylogeny.

**Fig. 2:**
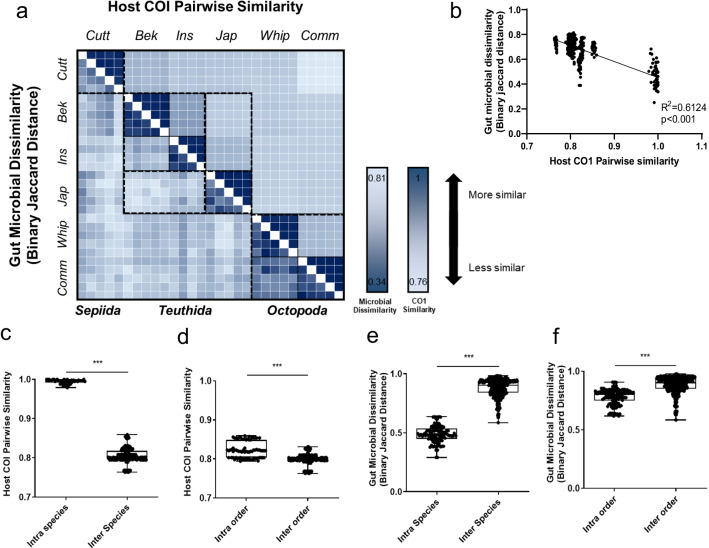
Association of host similarity and gut microbial dissimilarity. **a** Heatmap of gut microbial dissimilarity based on binary Jaccard distance metrics (left lower half) and host genetic relatedness based on COI gene similarity (right upper half). The range of colors indicates microbial dissimilarity or host COI gene dissimilarity: from light blue (highest gut microbial dissimilarity or lowest host COI gene dissimilarity) to dark blue (lowest gut microbial dissimilarity or highest host COI gene dissimilarity). **b** Linear regression analysis with the slope of the regression line for host COI gene similarity versus microbial dissimilarity.** c**,** d** Comparisons of intra- and inter-specific (**c**) and intra- and inter-order (**d**) host COI similarities. **e**–**f** Comparisons of intra- and inter-specific (**e**) and intra- and inter-order (**f**) microbial variation based on binary Jaccard distances. Asterisks indicate significant differences according to two-tailed Mann–Whitney U tests. *p < 0.05, **p < 0.01; ***p < 0.001. Abbreviations: Cutt, cuttlefish; Bek, beka squid; Ins, Inshore squid; Jap, Japanese flying squid; Whip, whiparm squid; Comm, common octopus

We then performed a heatmap analysis to investigate whether genetically similar hosts have similar gut microbial communities (Fig. 2a). Host genetic similarity was calculated using the COI gene sequence, while microbial dissimilarity was calculated using binary Jaccard distance. The host COI gene similarity and gut microbial dissimilarity showed a significant negative correlation in linear regression analysis (Fig. 2b), while intra-species/order variation was significantly lower than inter-species/order variation, both in COI similarity and binary Jaccard dissimilarity (Fig. 2c–f). Order *Octopoda* was found to be phylogenetically and morphologically heterogeneous from *Sepiida* and *Teuthoidea*. Interestingly, although the intra-order COI gene similarity of order *Octopoda* was not significantly different from other orders, the microbial intra-order distance was significantly lower (Additional file 1: Fig. S5).

**Fig. 3:**
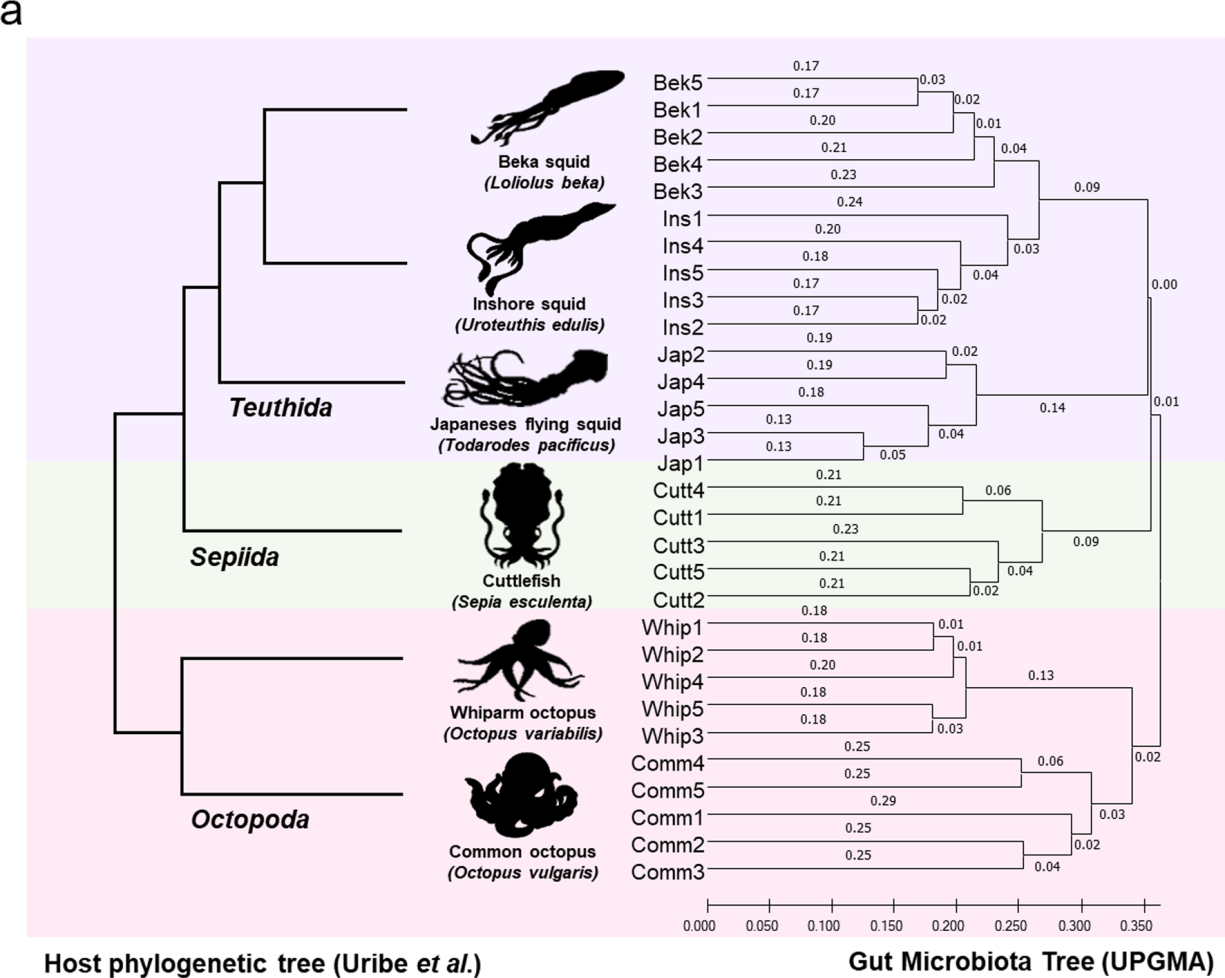
Phylosymbiotic host-gut microbiota assembly in cephalopods.** a** UPGMA-clustering dendrograms for cephalopod gut microbiomes based on unweighted UniFrac distances, compared with the host phylogenetic tree based on complete host mitochondrial genomes reported by Uribe et al. [21]. Species of respective orders of cephalopods are depicted with a specific background color. Abbreviations: Cutt, cuttlefish; Bek, beka squid; Ins, Inshore squid; Jap, Japanese flying squid; Whip, whiparm squid; Comm, common octopus

We evaluated whether there was a similar correlation between host phylogeny and gut microbial composition in cephalopods. Based on the phylogenetic tree using the complete mitochondrial genome described in previous study [21], we reconstructed the tree (host phylogeny tree) to contain only six species of cephalopods that we used. We also generated an unweighted-pair-group method with an arithmetic-mean (UPGMA) tree (i.e., a microbiota tree) to hierarchical clustering based on the gut microbial community composition of each cephalopod species (using binary Jaccard distance) (Fig. 3). Interestingly, each node of the cephalopod gut microbiota tree-shaped clades showed identical topologies to the host phylogeny tree in accordance with their host phylogeny.

A majority of OTUs were matched to the *Mycoplasma* and *Photobacterium* genera, which were regarded as the core taxa of the cephalopod gut microbiota (48.3% and 23.8%, respectively; Additional file 1: Fig. S4). Although OTUs belonging to these genera were differentially distributed according to host phylogeny, genus *Mycoplasma* was abundant in cuttlefish, Japanese flying squid, and whiparm octopus, while genus *Photobacterium* was predominant in beka squid and inshore squid. However, the limited taxonomic resolution means that an OTU-level analysis would be ineffective to explain detailed co-evolutionary histories between host and gut microbial species. Furthermore, sequences included in major OTUs are overestimated during taxonomic stratification, distorting the sequence distribution. To overcome these obstacles, we decomposed the OTUs assigned to identical genera (*Mycoplasma* and *Photobacterium*) and re-clustered the sequences into fine-scale units using nucleotide entropy by the minimum entropy decomposition (MED) method, which is an unsupervised oligotyping approach [22]. The OTUs belonging to *Mycoplasma* and *Photobacterium* were resolved into 228 oligotypes and distortion in the sequence distribution was reduced (Additional File 1: Fig. S6).

**Fig. 4:**
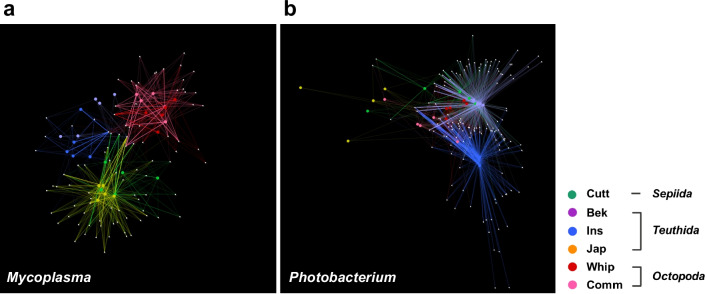
Network analyses of two core genera in cephalopod species, constructed by an unsupervised oligotyping approach. The networks of oligotypes belonging to **a** *Mycoplasma* and **b** *Photobacterium* were plotted. The edges connecting nodes representing cephalopod samples (large circles) to identified oligotypes in a particular sample are colored according to the host species (edge-weighted spring embedded model in Cytoscape v. 3.4.0). Abbreviations: Cutt, cuttlefish; Bek, beka squid; Ins, Inshore squid; Jap, Japanese flying squid; Whip, whiparm squid; Comm, common octopus

We performed network analysis using oligotypes to evaluate the distribution of the core taxa with better taxonomic resolution (Fig. 4). The distribution of the oligotypes among the hosts was consistent with the aforementioned results for the core OTUs and showed host-specific connections. In the case of *Mycoplasma*, oligotypes were divided into three sub-clusters according to host, namely cuttlefish and Japanese flying squid, beka squid and inshore squid, and whiparm octopus and common octopus. The majority of the *Photobacterium* oligotype nodes were connected to multiple hosts. There was also a striking difference in co-speciation patterns between *Mycoplasma* and *Photobacterium* in the oligotype-level phylogenetic analysis (Additional file 1: Fig. S7). In *Mycoplasma*, we found that most oligotypes colonized a single host species. Oligotypes assigned to *Photobacterium* that diverged earlier were found in multiple host species, whereas those that diverged more recently were host-specific. Detailed topological measures were calculated from MED network plots from *Mycoplasma* and (b) *Photobacterium*, were provided via Additional file 3: Table S2.

### The abundance of cephalopod core taxa is associated with host body size

We also conducted a beta-diversity analysis that gave weight to relative abundance using the Bray–Curtis dissimilarity indices (Additional file 1: Fig. S8). The plots were still clustered according to host species but not to host orders (Additional file 1: Fig. S8b-c). Furthermore, the relative abundance of core taxa differed between cephalopods belonging to the same order (Additional file 1: Fig. S4) meaning that there are other factors that determine the predominant taxa in addition to host phylogeny. *Mycoplasma* were found in significantly higher abundance in the common octopus and the Japanese flying squid. The whiparm octopus, which also belonged to *Octopoda*, had *Mycoplasma* levels more in common with cuttlefish (order *Sepiida*) than the common octopus. Beka squid and inshore squid had significantly lower *Mycoplasma* levels.

The abundance of *Mycoplasma* appears to be determined by the host’s body size rather than host phylogeny because the common octopus and Japanese flying squid had the largest body size among our samples. Indeed, a linear regression analysis showed that host body weight and *Mycoplasma* abundance were positively correlated analysis (Additional File 1: Fig. S9a), while *Photobacterium* predominated in smaller hosts (i.e., beka squid and inshore squid) with smaller body sizes. However, the correlation was not significant (Additional File 1: Fig. S9b).

### Host phylogeny, diet, and habitat shape the gut microbiota of mollusks

We next compared the gut microbiota of cephalopods and other mollusks to identify the relative contributions of various environmental, dietary, and phylogenetic factors that could influence microbial community composition (Table 1). We obtained data for the gut microbiomes of six mollusk species from public databases: the bone-eating snail (*Rubyspira osteovora,* *Bathymargarites* sp., and *Phymorhynchus* sp.) [23], the emerald sea slug (*Elysia chlorotica*) [24], the freshwater snail (*Planorbella trivolvis*) [25] the Hawaiian land snail (*Achatinella mustelina)* [26] of class *Gastropoda* and the oyster (*Crassostrea virginica*) [27] of class *Bivalvia*. A marine fish gut microbiome (62 species) [28] was also included in the analysis for comparison between mollusks and vertebrates.

**Table 1:** General informations for achieved data from previous studies

Host	Phylogeny	Habitat	Diet	References
Cephalopod	Invertebrate; Molluska; Cephalopoda	Seawater	Carnivore	This study
Bone-eat snail	Invertebrate; Molluska; Gastropoda	Seawater	Carnivore	Aronson et al. [23]
Emerald seaslug	Invertebrate; Molluska; Gastropoda	Seawater	Herbivore	Devine et al. [24]
Freshwater snail	Invertebrate; Molluska; Gastropoda	Freshwater	Herbivore, Omnivore	Hu et al. [25]
Hwaiian landsnail	Invertebrate; Molluska; Gastropoda	Terrestrial	Herbivore	O'Rorke et al. [26]
Oyster	Invertebrate; Molluska; Bivalvia	Seawater	Omnivore	King et al. [27]
Fish	Vertebrate	Seawater	-	Kim et al. [28]

**Fig. 5:**
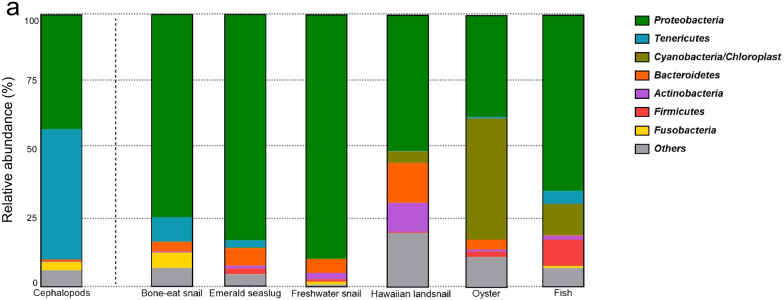
Gut microbial community structure of mollusks and fish. **a** Bar charts of the relative abundance of bacterial phyla in cephalopods, other mollusks, and fish. Only genera with a relative abundance of > 0.5% are shown; those with < 0.5% are classified as “Others.”

Each mollusk class and fish had a highly distinctive gut microbial composition (Fig. 5). Phylum *Tenericutes,* the core phylum of *Cephalopoda,* was found in the gut microbiota of cephalopods, the bone-eat snail, the emerald sea slug, and the fish. The cephalopods also had a significantly greater abundance of *Tenericutes* than any other group (Additional file 1: Fig. S10a). *Proteobacteria*, another core phylum of *Cephalopoda*, was observed in all groups but was significantly greater in both the emerald sea slug and freshwater snail, which are both freshwater *Gastropoda* (Additional file 1: Fig. S10b). At the genus level, *Mycoplasma* and *Photobacteria* were only predominant in the cephalopod species (Additional file 1: Fig. S10c, d).

**Fig. 6:**
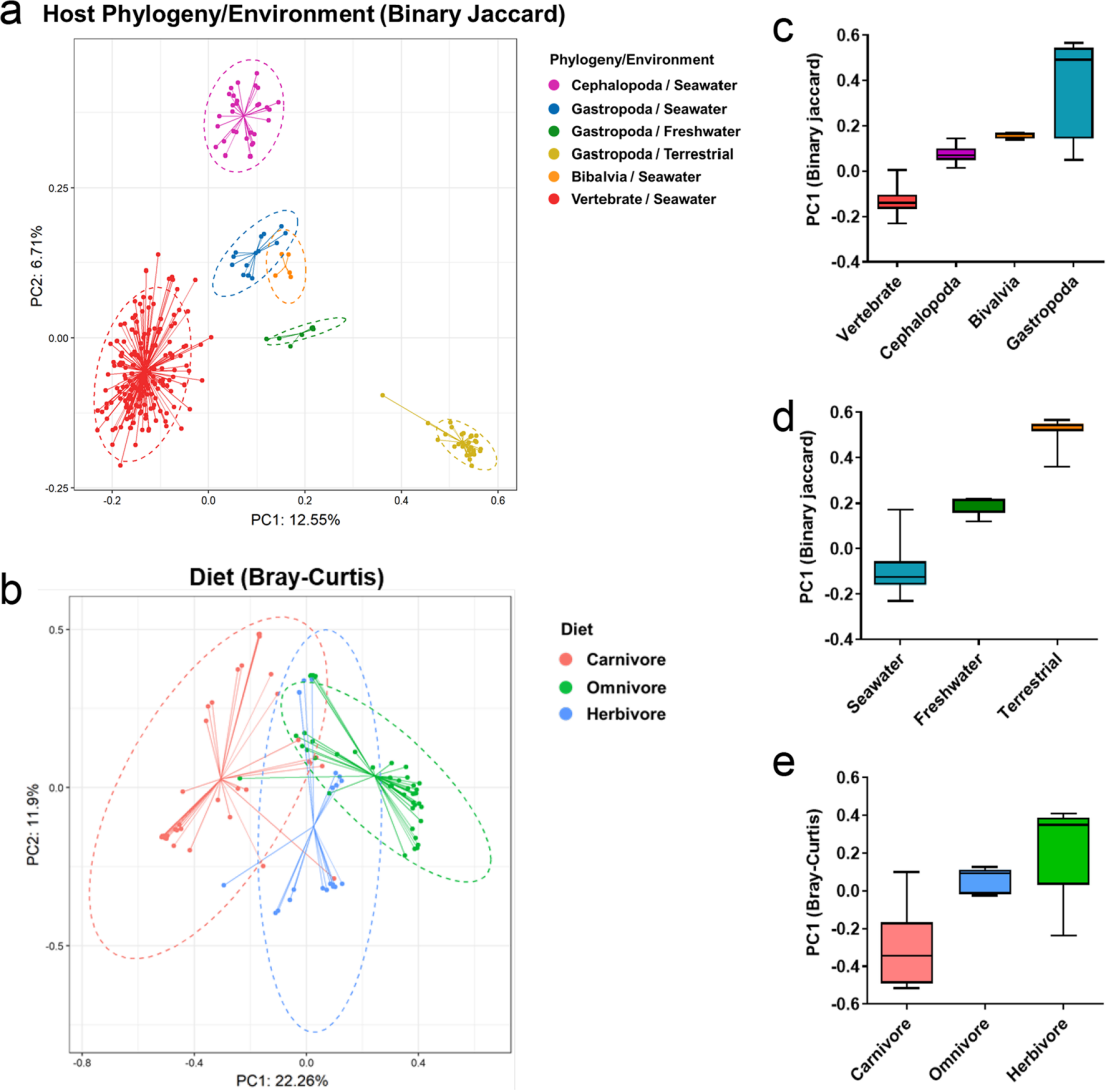
Ecological characteristics of global mollusk and fish gut microbiomes. **a**, **b** PCoA ordination of the microbial community of mollusks and fish with **a** binary Jaccard and **b** Bray–Curtis indices. The different colors represent **a** host phylogeny, habitat, and **b** diet group. Centroids and ellipses for each group are also shown. **c**–**e** Boxplot diagram of the PC1 for each **a** host phylogeny/habitat and **b** diet group. The letters above the whisker indicate significant differences (*p* < 0.05) among groups (Mann–Whitney U test)

As mentioned earlier, cephalopods have a closed circulation system [18], advanced nervous systems [19] and well-differentiated digestive system [20]. These are features more commonly found in vertebrates, so we expected the cephalopod microbiome to be similar to vertebrate microbiomes. We compared the microbiomes of mollusks belonging to *Cephalopoda*, *Gastropoda*, *Bivalvia*, and marine fish (vertebrates) using a beta-diversity analysis (Fig. 6a). Surprisingly, the cephalopod microbiomes made a cluster that was distinctive from the other mollusks and also from the fish, which appears to suggest that the microbiomes of cephalopods are significantly influenced by host phylogeny.

*Gastropoda* showed greater intra-class variation than either *Cephalopoda* or *Bivalvia* (Fig. 6c). We therefore surmised that host heterogeneity, habitat, or the diets of the *Gastropoda* species in our dataset would lead to large microbiome variation. Indeed, PCoA plots were made clusters according to the host’s habitat (marine, freshwater, terrestrial). We also noticed that the microbial composition of marine gastropods was more similar with bivalves and cephalopods than terrestrial gastropods, which seems to indicate that the host’s habitat is a key factor in determining the microbiome composition of mollusks (Fig. 6d).

Diet is also a major shaping factor of microbiota. To evaluate the effect of diet on mollusk microbiomes, we performed beta-diversity subgroup analysis using the mollusk dataset. Fish data were excluded from this analysis as the dietary information was unclear. Unexpectedly, binary Jaccard analysis found that the PCoA plots of mollusk microbiomes were not distinguished by their host’s diet (Additional File 1: Fig. S11), while *Cephalopoda* and terrestrial *Gastropoda* made separate clusters. We hypothesized that the effect of diet on microbiome composition was diluted because the overall microbial composition was strongly influenced by the host’s phylogeny and habitat. The effect of diet on the mollusk microbiome became clear with the Bray–Curtis analysis, which is a weighted method (Fig. 6b). In PCoA analysis, the plots of carnivores, omnivores, and herbivores formed distinct clusters, with omnivores plotted between carnivores and herbivores (Fig. 6e). Accordingly, we concluded that the microbial community in mollusks is determined by host phylogeny and habitat, while diet can determine the abundance of major bacteria.

## Discussion

There are relatively few studies that have explored the cephalopod gut microbiome. The gut microbiome of *Octopus mimus* was investigated using a 16S rDNA clone library [29] while the first cephalopod gut microbial analysis using next-generation sequencing was performed on free-living and captive *Octopus minor* paralarvae [30]. The microbial composition of the digestive tract, gills, and skin microbiome of *Sepia officinalis* was demonstrated in a recent study [31]. In this study, we characterized the microbiomes of six free-living cephalopod species (cuttlefish, beka squid, inshore squid, Japanese flying squid, common octopus, and whiparm octopus) belonging to three orders (*Teuthida*, *Speiida,* and *Octopoda*) and compared them with the microbiomes of other mollusks and marine fish. To the best of our knowledge, our study is the first multi-species analysis of cephalopod microbiomes.

Each of the three cephalopod orders we sampled had very distinctive features. *Sepiida* and *Teuthoidea* have an internal shell inside the body and have ten legs, including two tentacles; *Octopoda*, by contrast, have no internal shell and only eight legs. The pupil structure of *Sepiida* is w-shaped, while *Teuthoidea* have round pupils and *Octopoda* rectangular pupils. Compared with other cephalopods, *Octopoda* have a more differentiated digestive system, an advanced nervous system, and higher intelligence which could also possibly affect the microbiome. Such morphological characteristics can affect the microbiome by themselves and have the potential to affect the host behavior, hunting method, and diet.

Based on our comparative analysis of 16S ribosomal RNA (rRNA) gene sequences obtained using Illumina MiSeq sequencing, we found that the *Mycoplasma* and *Photobacterium* genera were the core taxa found in cephalopod gut microbiota. These genera are also found in the digestive tracts of wild Chilean octopus [29], aquacultured common octopus [32] and cuttlefish [31].

*Mycoplasma* is an obligate parasitic bacterial group and is a key component in the gut microbiome of many marine animals such as the Norway lobster [33], jellyfish [34], and various fish species [35–38]. Their roles in the intestinal ecosystems of terrestrial vertebrates are typically recognized as pathogenic or opportunistic [39–41]. By contrast, marine vertebrates, especially salmon, are known to have a symbiotic relationship with *Mycoplasma* [15, 38, 42]. A metagenome-assembled genome study of gut microbial *Mycoplasma* in salmonoids revealed that the intestinal commensal *Mycoplasma* actively metabolizes using ammonia [38]. However, little is known about their role in invertebrate microbiomes, other than a study reporting a potential symbiotic relationship in scorpions [43]. We suspect that cephalopods may also have symbiotic relationships with gut *Mycoplasma* through ammonia metabolism, as in the case of salmonoids, because cephalopods are both carnivorous and ammonotelic. To further investigate commensalism in cephalopods and *Mycoplasma*, a shotgun metagenomic study will be necessary.

*Photobacterium* is well known for its bioluminescent properties [44] and its pathogenicity [45, 46]; however, their phylogeny and taxonomy are not clearly elucidated [47]. Members of *Photobacterium* show ecological diversity and include taxa that are symbiotic [48–50] or parasitic [51, 52] with marine animals, those that are free-living in seawater [53] and saline lake water [54], and even those in high pressure habitats [55]. Bioluminescence is a common feature of many genera in *Vibrionaceae,* and *Photobacterium* is one of the most extensively studied groups [56, 57]. In this study, *Photobacterium* was particularly abundant in beka squid (58.0%) and inshore squid (75.9%), and in members of the sub-order *Myopsida,* such as the Hawaiian bobtail squid (*Euprymna scolopes*). The Hawaiian bobtail squid is famous for its light-associated symbiosis and symbiont-specific immune tolerance with the bioluminescent bacterium *Aliivibrio fischeri* [16, 58], which was once assigned to the *Photobacterium* genus [47].

Although beka squid and inshore squid are not bioluminescent, the predominance of *Photobacterium* in *Myopsida* hosts suggests that there is a general symbiotic relationship between *Myopsida* hosts and *Vibrionaceae* bacteria. *Photobacterium* is also known to have a symbiotic relationship with some fish species as it can decompose chitin within the intestinal tract [59]. Chitin is the main component of crustacean shells. Since it is known that smaller cephalopods prefer crustaceans as prey [60], the high *Photobacterium* abundance in *Myopsida* gut microbiota might be related to their diet.

The COI gene is a mitochondrial housekeeping gene that is widely used in animal identification and phylogenetic research [61, 62]. We sequenced the COI gene from the flesh of cephalopod hosts and used the resulting data to identify cephalopod samples and to measure pairwise similarities between the samples. However, the COI gene-based tree we constructed did not match the actual cephalopod phylogeny in all the construction methods that we tried (neighbor-joining, maximum likelihood, and maximum parsimony (data not shown)). We can speculate that the cause of the discrepancy between the COI-based tree and the actual phylogeny is that we did not study a sufficient number of cephalopod hosts. Thus, the host phylogeny tree in our study was only used to compare the topology with the hierarchical microbiota tree without statistical analysis.

In microbial community analyses by 16S amplicon sequencing, the sequences are typically clustered into OTUs based on similarity, with a typical threshold of 97%. This clustering process is beneficial for downstream analyses. However, when regarding the operational definition of a species, 3% dissimilarity is only a rough approximation. There is a risk, therefore, that closely related species could be identified as a single taxonomic unit in the clustering process.

Furthermore, OTU-based analyses showed a limited resolution for analyses below the genus level. However, the MED method overcomes some of the limitations of the OTU-based approach as it provides a computationally efficient means to partition marker gene datasets into MED nodes, which represent homogeneous OTUs. We used the MED approach to perform a network analysis at the within-genus level. The oligotyping analysis revealed different co-evolutionary histories between two major cephalopod species. The distribution of the oligotypes of *Mycoplasma* was concentrated with host-specific colonization, although a large number of *Photobacterium* oligotypes were found in cephalopod species. Based on these results, *Mycoplasma* colonization in cephalopods was found to be frequently related to host-specific evolution or biological activities, while *Photobacterium* colonized cephalopods more broadly as interactions with *Photobacterium* might be essential for the survival or adaptation of cephalopod species to their habitats. This finding agrees with a microbiome study of the gut of Atlantic cod [49], which found that *Vibrionaceae*, including *Photobacterium*, is found in the vast majority of both cod and other marine carnivore fish.

The binary Jaccard distance matrix is calculated according to the presence or absence of bacterial taxa in the community and the abundance of taxa is not reflected in the result [63]. This method is effective in analyzing the overall composition of the microbial community, including rare taxa, although it does not reflect the abundance of each bacterial taxa. Therefore, it will be more efficient to use a matrix that reflects abundance, such as the Bray–Curtis dissimilarity [64]. Intra-host order dissimilarity was observed in the Bray–Curtis analysis, but not in binary Jaccard analysis, which means that the host phylogeny determines the composition of cephalopod gut microbiota but not the abundance of each bacterial type.

We found that host body weight is associated with cephalopod gut bacterial abundance. *Mycoplasma* abundance showed a significant correlation with host body weight, and the abundance of *Photobacterium* tended to decrease as body weight increased. It is well known that cephalopods, as predators, consume different types of prey depending on their body size [65]. Juvenile or smaller cephalopods prefer a crustacean diet [60] while larger cephalopods consume a variety of prey and sometimes engage in cannibalism [66–68]. Cephalopod diet may be correlated with the abundance of gut *Mycoplasma*. Given that *Mycoplasma* is known to actively metabolize ammonia in the intestine [38], larger cephalopods are likely to produce more ammonia. An interesting follow-up study could investigate the association between prey found within the cephalopods and their gut microbiota. Regarding *Photobacterium*, beka squid and inshore squid (order *Teuthida*) have a small body size and a higher abundance of *Photobacterium*. However, the whiparm octopus also has a small body size, but *Photobacterium* is not the predominant taxa. Instead, the Japanese flying squid (order *Teuthida*) had *Photobacterium* as a core taxon. Accordingly, *Photobacterium* abundance is thought to be strongly influenced by host phylogeny when compared with *Mycoplasma*.

To the best of our knowledge, our study is the first to conduct a comparative analysis of cephalopod and mollusk gut microbiota. We identified three factors that influence the gut microbiota of cephalopods and mollusks: host phylogeny, habitat type, and diet. All mollusks had very different microbiota to fish, regardless of their diet or habitat, which means that host phylogeny is an important factor in shaping their microbiota. Marine *Gastropoda* such as the bone-eat snail, and emerald sea slug had a similar microbial composition to freshwater *Gastropoda,* despite differences in habitat. Cephalopods are also a good illustration of the associations between mollusk gut microbiomes and their host phylogeny. We found that habitat is also a very strong factor in determining mollusk microbiomes. Mollusks were clustered according to their habitat in beta-diversity analysis, particularly the microbiota of terrestrial and marine mollusks. The relationship between the host’s habitat and gut microbiome has been extensively studied [69, 70]. Marine mammals generally have higher *Fusobacteria* abundance and lower *Bacteroidetes* abundance in their microbiomes than terrestrial mammals [71, 72], while the microbiota of fish is more strongly shaped by habitat than diet or host phylogeny [28]. Finally, our Bray–Curtis analysis showed that mollusk gut microbiota was distinguished by diet. The bone-eat snail had a similar microbiome to cephalopods, despite being a gastropod. In addition, herbivore marine mollusks had similar microbial communities to freshwater snails which are also herbivore.

Our analysis has several limitations. First, there were not a sufficient number of samples to adequately compare each factor. In the future, more diverse studies on mollusk microbiomes are necessary to reinforce data through further analysis, including more varied animal microbiome samples. Second, the sequencing platform and data regions used in the analysis were not unified. This hinders the application of the same analytical method to the processing of each dataset and makes analysis of the OTU level impossible. We also did our best to reduce bias from the sequencing platform and region. It is well known that alpha diversity is strongly influenced by the sequencing platform and region, and beta-diversity is strongly influenced by 16S/shotgun and analytic methods [73]. Therefore, we did not include the alpha diversity analysis results of the downloaded data in our study and unified all the analysis methods and parameters. Our study did not include shotgun metagenomic data.

Other topics we have expressed an interest in are alpha diversity and sexual dimorphism. Invertebrates are known to have very simple gut microbiota compared with vertebrates [9–11], so we wondered whether the “complexity” of the microbiome in cephalopods, which have many biological characteristics of vertebrates, would be more similar to vertebrates or invertebrates. By conducting beta-diversity analysis, we determined that the overall composition of the cephalopod microbiome is a unique ecosystem, which differs from vertebrates and other mollusk groups. However, since the sequencing platform and sequencing region of the data used for comparison were different, we could not use the same bioinformatic analysis methods, making it impossible to compare alpha diversity A single study containing cephalopods, other mollusks, and vertebrate microbiome sequencing data is needed in order to conduct an alpha diversity comparison. In our study, we expected the cephalopod gut microbiota to differ between sexes based on differences in growth rate, body size, diet, and space niche between male and female octopuses [74, 75]. However, we found no significant differences in the gut microbial composition between the sexes (data not shown). It is highly likely, however, that the result does not reflect the real world because the proportion of females in our cephalopods samples was too low to conduct a robust statistical analysis. Therefore, a re-analysis with sufficient proportions of male and female specimens is required to evaluate the effect of sexual dimorphism on the cephalopod intestinal microbiome.

We found that features of the cephalopod and mollusk gut microbial communities were relatively similar to the common features of the vertebrate gut microbiota, which are also affected by host phylogeny [76], evolutionary divergence time [77], living environment [5], and diet [78]. The shared characteristics of their microbiomes suggest that insights from studies of the vertebrate gut microbiota can be applied to invertebrate studies, which can help establish future directions for invertebrate gut microbiome research. New findings based on invertebrate gut microbiome studies can then have the potential to be applied to vertebrate and human research. For example, *Mycoplasma* and *Photobacterium* are predominant in cephalopods. Thus, cephalopods will be a very useful tool for studying the interactions between the vertebrate host and these genera [12]. Indeed, *Mycoplasma* is commensal bacteria that are important for the health of farmed salmon [15, 79], although their commensal mechanism is largely unknown. In our data, *Mycoplasma* accounted for over 97% of the gut microbial community of the common octopus. Therefore, the octopus would be a very useful model for examining the symbiotic relationship between *Mycoplasma* and marine animals. Furthermore, the knowledge gained through modulation of diet, habitat, and host genetic factors to mollusks can be applied to studies of vertebrate microbiomes.

## Conclusions

In summary, we have performed the first comparative analysis of the cephalopod gut microbiota using a high-throughput sequencing approach. We have revealed that each *Cephalopoda* species that we studied has a unique gut microbiota. Both *Mycoplasma* and *Photobacterium* were core taxa in the gut microbiota of cephalopods. Furthermore, we found that the cephalopod gut microbial community composition was determined by host phylogeny, which is also an important determinant of the gut microbiota of marine mollusks. Diet and habitat also contributed to the composition of mollusk gut microbiota.

## Materials and methods

### Sampling

Cuttlefish, beka squid, inshore squid, Japanese flying squid, common octopus, and whiparm octopus were captured from the offshore waters surrounding the Republic of Korea, with five individuals sampled for each cephalopod species. All samples were directly transferred to the laboratory before being sacrificed using an anesthetic. The dorsal mantle length and weight of each individual were determined before the samples were dissected to remove the stomach, cecum, and other digestive organs. Detailed metadata for the cephalopod samples are presented in Additional File 4: Table S3.

### Identification of cephalopod hosts by cytochrome oxidase I sequencing

The cephalopod subjects were initially subjected to basic taxonomic identification based on morphological characteristics. For a more detailed identification, genomic DNA was aseptically extracted from the flesh of the specimens. A fragment of each tissue sample was then suspended in 750 ml of lysis buffer and homogenized by FastPrep-24 (MP Biomedicals, Santa Ana, CA, USA) with glass beads (0.5 mm diameter) for 45 s at 5.0 m/s. After lysis, standard phenol–chloroform DNA extraction was performed. The DNA extracts was PCR-amplified using cytochrome c oxidase subunit I (COI) primers designed for diverse metazoan invertebrates. PCR products were purified using the QIAquick PCR Purification Kit (Qiagen, Hilden, Germany) following standard protocol, and were bidirectionally sequenced using an automated DNA analyzer system (PRISM 3730XL DNA Analyzer; Applied Biosystems, Foster City, CA, USA) and the BigDye Terminator Cycle Sequencing Ready Reaction Kit (Applied Biosystems). The sequence fragments were assembled using SeqMan (DNASTAR).

The assembled COI gene sequences were then compared with other COI gene sequences in the nucleotide collection (nr/nt) in GenBank by a BLAST search (Additional File 2: Table S1).

### DNA extraction and sequencing of bacterial 16S rRNA genes

The cecum was primarily used to investigate the gut microbial communities of the cephalopod samples. The cecal contents of the dissected cecal samples were also collected and pooled with cecum. In order to maximize microbial cell lysis for DNA extraction, the cecum and cecal contents were homogenized by shaking them in a sterile screw tube containing zirconia beads (2.3 mm, 0.1 mm diameter) and glass beads (0.5 mm diameter) for 50 s using FastPrep-24 (MP Biomedical). After lysis, the microbial DNA from the homogenized gut samples were extracted using the Qiagen DNA Stool Mini Kit (Qiagen). The V3-4 hypervariable region of the 16S rRNA gene was amplified with the primers 341F (5′-CCTACGGGNGGCWGCAG-3′) and 805R (5′GACTACHVGGGTATCTAATCC-3′), and four independently amplified products for each sample were pooled and purified using the QIAquick PCR Purification Kit (Qiagen) in order to minimize bias. We used negative controls in the DNA extraction, PCR, and purification processes to control the contamination generated during the experiment. No contamination was detected during the experiment. DNA libraries were prepared using the Nextera XT DNA Library Preparation Kit for the Illumina MiSeq platform (Illumina, San Diego, CA, USA) and were then sequenced by certified service provider (Macrogen, Seoul, Korea) using the Illumina MiSeq platform with 2 × 300 bp reads, following the manufacturer’s instructions.

### Sequence analysis

The raw 16S rRNA sequence data were processed using QIIME 1.9.1. Paired-end sequence reads were assembled with default parameters and minimally quality filtered, with a Phred quality score threshold of 20. Data were then error-filtered using USEARCH (a de novo chimera removal algorithm). High-quality sequence reads were assigned to OTUs by an open-reference OTU picking protocol using the QIIME toolkit, where the UCLUST, OTU picking algorithm was applied to search sequences against the Greengenes reference database from August 2013 at a 97% sequence similarity at a 97% sequence similarity threshold. A representative sequence for each OTU was aligned with the Greengenes reference using PyNAST. For the bacterial taxonomic assignment, an RDP classifier (Version 2.3; https://rdp.cme.msu.edu/classifier/classifier.jsp) was used, with a confidence value threshold of 80%. An even-depth rarefied OTU table matrix (6000 sequences) was constructed. Sequences belonging to the *Mycoplasma* and *Photobacterium* genera were clustered with MED for sensitive discrimination of closely related organisms.

### Network-based analysis of* Mycoplasma* and* Photobacterium*

Network maps of *Mycoplasma* and *Photobacterium* were generated using QIIME and were visualized using Cytoscape (version 3.4.0), while the even-depth rarefied MED tables were constructed with *Mycoplasma* and *Photobacterium* and converted to Cytoscape format using a QIIME script (*make_otu_network.py*) [80, 81]. In the converted MED network maps, samples and MEDs represented nodes of the network and these nodes were connected by edges, indicating the abundance of the MED in the samples. Edge-weighted spring embedded models were derived for network arrangement. Topological analysis of MED network was performed using Cytoscape and MCODE plug-in toolkit [82].

### Comparison of gut microbiomes of cephalopods and various animal

Sequence data for the sea slug (*Elysia chlorotica*) and Eastern oyster (*Crassostrea virginica*) gut microbiomes were obtained from the MG-RAST server (mgp561 and mgp1994, respectively; http://metagenomics.anl.gov) [24, 27], while sequence data for the Hawaiian land snail (*Auriculella ambusta*) and freshwater snail (*Planorbella trivolvis*) gut microbiomes was downloaded from NCBI Sequence Read Archive (SRP047488 and SRP268119, https://www.ncbi.nlm.nih.gov/sra) [83]. Sequenced data for the bone-eat snail were downloaded from Dryad Digital Repository [23] (http://dx.doi.org/10.5061/dryad.5h1q1). Detailed information about the downloaded dataset was described in Additional file 4: Table S3. Since the targeted region and the applied sequencing technologies varied between experiments, we assigned taxonomic characteristics against the identical reference database using an RDP classifier. After unaligned sequences were discarded, an even-depth rarefied OTU table was generated and used for further analyses. Non-phylogenetic distance metrics (binary Jaccard and Bray–Curtis dissimilarities) were calculated and visualized by a 2D PCoA.

### Statistical analysis

The alpha diversity of microbial community was assessed using observed species, Chao1, Shannon, and Faith’s PD indices. The beta diversity was calculated using binary Jaccard and Bray–Curtis indices using QIIME pipeline. The host COI gene similarity was calculated by pairwise comparison between COI gene sequences and gut microbial dissimilarity was extracted from binary Jaccard distance matrix. The group comparison was analyzed using the Mann–Whitney U test and visualized with box and whisker with individual plots. Boxplot centerline represents the median (50th percentile). The top and bottom hinges represent 75th and 25th percentiles, respectively. The upper and lower whiskers correspond to the highest and lowest data points. The correlation analysis was performed with linear regression.

## Supplementary Information

**Additional file 1**. **Fig. S1:** Alpha diversity indices of the cephalopod gut microbiota. (a) Number of observed species. (b) Chao1 index. (c) Shannon diversity. (d) Faith’s PD. The letters above the whisker indicate significant differences (*p* < 0.05) among groups (Mann–Whitney U test). Abbreviations: Cutt, cuttlefish; Bek, beka squid; Ins, Inshore squid; Jap, Japanese flying squid; Whip, whiparm squid; Comm, common octopus. **Fig. S2:** Rarefaction curves of the abundance-based coverage estimation against the cumulative number of identified OTUs. Coverage plots are generated with the number of observed species. The line colors in the rarefaction curves represent the host species. Abbreviations: Cutt, cuttlefish; Bek, beka squid; Ins, Inshore squid; Jap, Japanese flying squid; Whip, whiparm squid; Comm, common octopus. **Fig. S3:** Gut microbial compositions of cephalopods. Bar charts of the relative abundance of bacterial phyla in six cephalopod species as well as the overall gut microbial composition of cephalopods. Only phyla with a relative abundance of > 1% are shown; those with an abundance of < 1% are classified as “Others.” Abbreviations: Cutt, cuttlefish; Bek, beka squid; Ins, Inshore squid; Jap, Japanese flying squid; Whip, whiparm squid; Comm, common octopus. **Fig. S4:** Distribution of core genera of cephalopods. Boxplot diagram of (a) Mycoplasma, (b) Photobacterium, (c) Alivibrio, (d) Acrobacter, and (e) Psychrilyobacter. The letters above the whisker indicate significant differences (*p* < 0.05) among groups (Mann–Whitney U test). Abbreviations: Cutt, cuttlefish; Bek, beka squid; Ins, Inshore squid; Jap, Japanese flying squid; Whip, whiparm squid; Comm, common octopus. **Fig. S5:** Comparisons of intra-order host COI similarity and microbial variation of Octopoda and other orders. Host similarity was calculated with pairwise COI sequence comparison. Microbial variation was calculated based on binary Jaccard distance. Asterisks indicate significant differences according to two-tailed Mann–Whitney U tests. *p < 0.05, **p < 0.01; ***p < 0.001. **Fig. S6.** Distributions of *Photobacterium* and *Mycoplasma* OTUs and oligotypes in cephalopod gut microbiomes. The distributions of 97% clustered OTUs assigned to *Photobacterium* and *Mycoplasma* (a–b) are compared with re-clustered oligotypes and generated using the MED pipeline with aligned sequence reads that originally matched to *Photobacterium* and *Mycoplasma* by the QIIME 1.9.1 pipeline. **Fig. S7:** Phylogenetic trees of *Photobacterium* and *Mycoplasma* based on oligotypes (maximum likelihood tree with 1000 bootstrap replicates and the GTR + Gamma model). Bar graphs are color-coded to show the proportions of oligotypes assigned to *Photobacterium* (a) and *Mycoplasma* (b) in each cephalopod species. Abbreviations: Cutt, cuttlefish; Bek, beka squid; Ins, Inshore squid; Jap, Japanese flying squid; Whip, whiparm squid; Comm, common octopus. **Fig. S8.** Beta-diversity analysis for cephalopod species using the Bray–Curtis index. Principal coordinates analysis (PCoA) of Bray–Curtis between cephalopod samples. The colors of the dots in the PCoA represent the host cephalopod species and their orders. (b–c) Comparisons of intra- and inter-specific (b) and intra- and inter-order (c) microbial variation based on the Bray–Curtis dissimilarity. Asterisks indicate significant differences according to two-tailed Mann–Whitney U tests. *p < 0.05, **p < 0.01; ***p < 0.001. Abbreviations: Cutt, cuttlefish; Bek, beka squid; Ins, Inshore squid; Jap, Japanese flying squid; Whip, whiparm squid; Comm, common octopus. **Fig. S9.** Linear regression analysis with the slope of the regression line. Correlation between body weight and relative abundance of *Mycoplasma* (a) was positive and significant, but body weight and relative abundance of *Photobacterium* (b) was negative but not significant. **Fig. S10.** Boxplot diagram of the relative abundance for the phyla (a) Tenericutes, (b) Proteobacteria, and genera (c) Mycoplasma, and (d) Photobacterium. The letters above the whisker indicate significant differences (*p* < 0.05) among groups (Mann–Whitney U test). **Fig. S11.** Beta-diversity analysis for mollusk and fish using the binary Jaccard and Bray–Curtis indices. PCoA plots with (a) binary Jaccard and (b) Bray–Curtis indices show different distribution patterns. The colors of the dots, centroids and ellipses in the PCoAs represent the group that each host belongs to.

**Additional file 2**. **Supplementary Table S1**. Basic information on the cephalopod hosts.

**Additional file 3**. **Supplementary Table S2**. Topological measures in oligotypes-based network analyses.

**Additional file 4**. **Supplementary Table S3**. Basic information on the acheived from previous studies.

**Additional file 5**. Code and Scripts.

## Data Availability

The newly generated 16S rRNA sequence datasets are available in the European Nucleotide Archive (ENA) of EMBL-EBI under the accession number PRJEB27490. The cytochrome oxidase subunit 1 (CO1) gene sequences used for identifying host species have been submitted to NCBI GenBank (https://www.ncbi.nlm.nih.gov/genbank) under accession numbers MH542436-MH542464 (under the title “Factors shaping invertebrates gut microbiota: host phylogeny, habitat, and diet are involved in shaping of gut microbiota of *Cephalopoda*, *Mollusca*”).

## Code availability

No custom codes were used. Software and packages, their versions, relevant specification and parameters are stated in Additional file 5: Code and Scripts.
