# Supplementary material for: Host phylogeny, habitat, and diet are main drivers of the cephalopod and mollusk gut microbiome
Source: Anim Microbiome. 2022 May 8;4:30. doi: 10.1186/s42523-022-00184-x (PMC9082898; doi:10.1186/s42523-022-00184-x)
Supplement: Supplementary file 1 — Additional file 1. Fig. S1: Alpha diversity indices of the cephalopod gut microbiota. (a) Number of observed species. (b) Chao1 index. (c) Shannon diversity. (d) Faith’s PD. The letters above the whisker indicate significant differences (p < 0.05) among groups (Mann–Whitney U test). Abbreviations: Cutt, cuttlefish; Bek, beka squid; Ins, Inshore squid; Jap, Japanese flying squid; Whip, whiparm squid; Comm, common octopus. Fig. S2: Rarefaction curves of the abundance-based coverage estimation against the cumulative number of identified OTUs. Coverage plots are generated with the number of observed species. The line colors in the rarefaction curves represent the host species. Abbreviations: Cutt, cuttlefish; Bek, beka squid; Ins, Inshore squid; Jap, Japanese flying squid; Whip, whiparm squid; Comm, common octopus. Fig. S3: Gut microbial compositions of cephalopods. Bar charts of the relative abundance of bacterial phyla in six cephalopod species as well as the overall gut microbial composition of cephalopods. Only phyla with a relative abundance of > 1% are shown; those with an abundance of < 1% are classified as “Others.” Abbreviations: Cutt, cuttlefish; Bek, beka squid; Ins, Inshore squid; Jap, Japanese flying squid; Whip, whiparm squid; Comm, common octopus. Fig. S4: Distribution of core genera of cephalopods. Boxplot diagram of (a) Mycoplasma, (b) Photobacterium, (c) Alivibrio, (d) Acrobacter, and (e) Psychrilyobacter. The letters above the whisker indicate significant differences (p < 0.05) among groups (Mann–Whitney U test). Abbreviations: Cutt, cuttlefish; Bek, beka squid; Ins, Inshore squid; Jap, Japanese flying squid; Whip, whiparm squid; Comm, common octopus. Fig. S5: Comparisons of intra-order host COI similarity and microbial variation of Octopoda and other orders. Host similarity was calculated with pairwise COI sequence comparison. Microbial variation was calculated based on binary Jaccard distance. Asterisks indicate significant differences according to [file 42523_2022_184_MOESM1_ESM.docx]

Supplementary Figures for

**Host phylogeny, habitat, and diet are main drivers of the cephalopod and mollusk gut microbiome**

Woorim Kang,^†^ Pil Soo Kim,^†^ Euon Jung Tak, Hojun Sung, Na-Ri Shin, Dong-Wook Hyun, Tae Woong Whon, Hyun Sik Kim, June-Young Lee, Ji-Hyun Yun, Mi-Ja Jung and Jin-Woo Bae*

Department of Life and Nanopharmaceutical Sciences and Department of Biology, Kyung Hee University, Seoul 130-701, Korea

^†^These authors contributed equally to this study.

*Author for correspondence: Jin-Woo Bae. Tel.: +82 2 961 2312. Fax: +82 2 961 9155. E-mail: baejw@khu.ac.kr

**
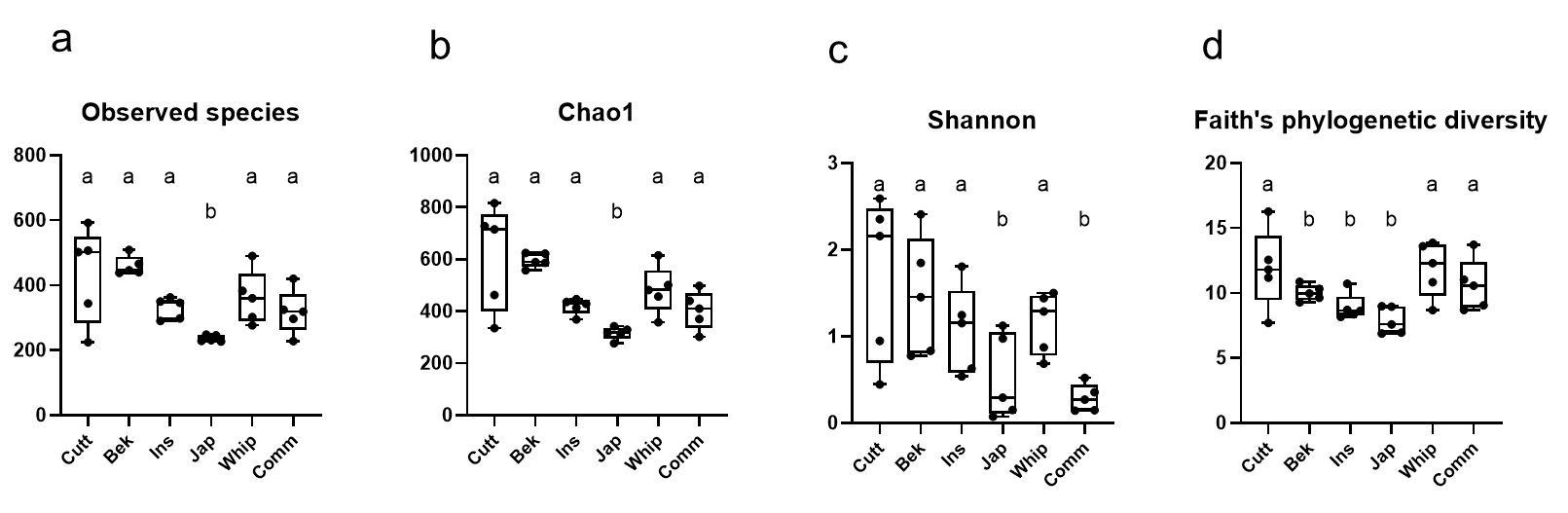
**

**Supplementary Fig. S1.** Alpha diversity indices of the cephalopod gut microbiota. (a) Number of observed species. (b) Chao1 index. (c) Shannon diversity. (d) Faith’s PD. The letters above the whisker indicate significant differences (p < 0.05) among groups (Mann-Whitney U test). Abbreviations: Cutt, cuttlefish; Bek, beka squid; Ins, Inshore squid; Jap, Japanese flying squid; Whip, whiparm squid; Comm, common octopus.


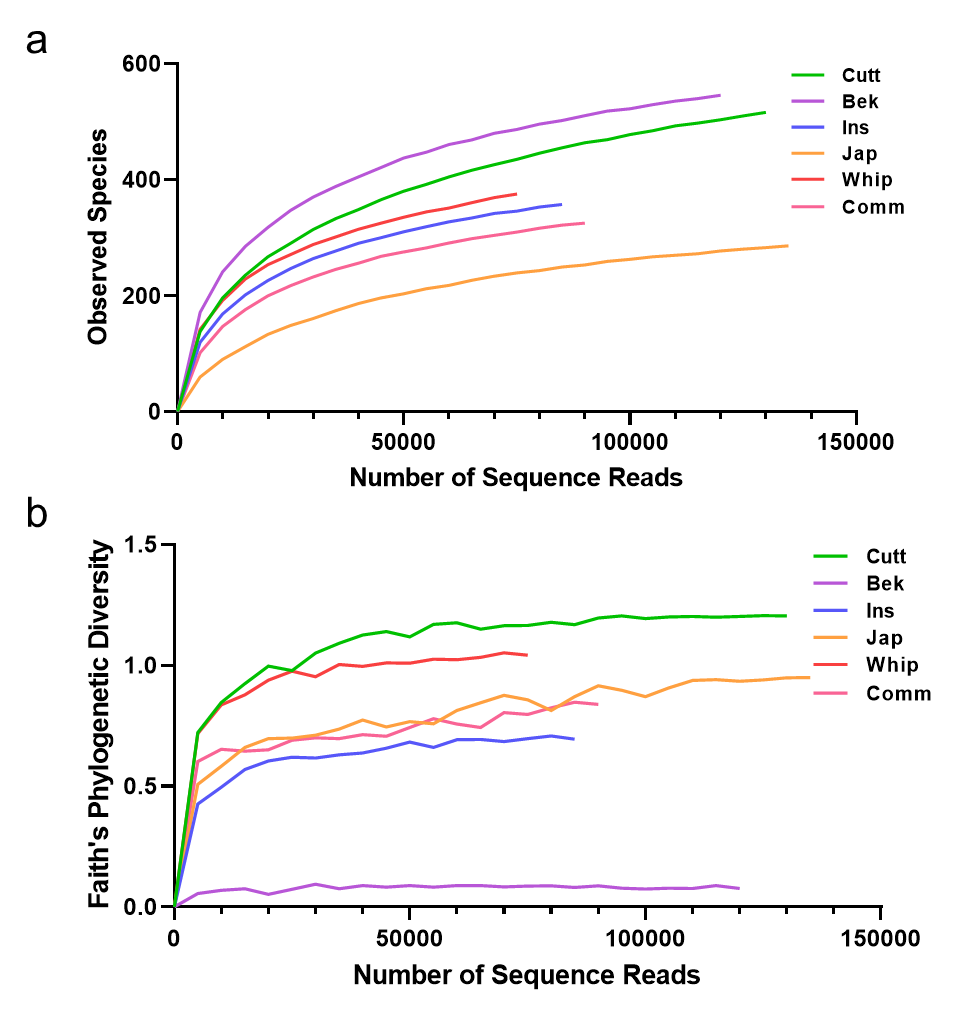


**Supplementary Fig. S2.** Rarefaction curves of the abundance-based coverage estimation against the cumulative number of identified OTUs. Coverage plots are generated with the number of observed species. The line colors in the rarefaction curves represent the host species. Abbreviations: Cutt, cuttlefish; Bek, beka squid; Ins, Inshore squid; Jap, Japanese flying squid; Whip, whiparm squid; Comm, common octopus.


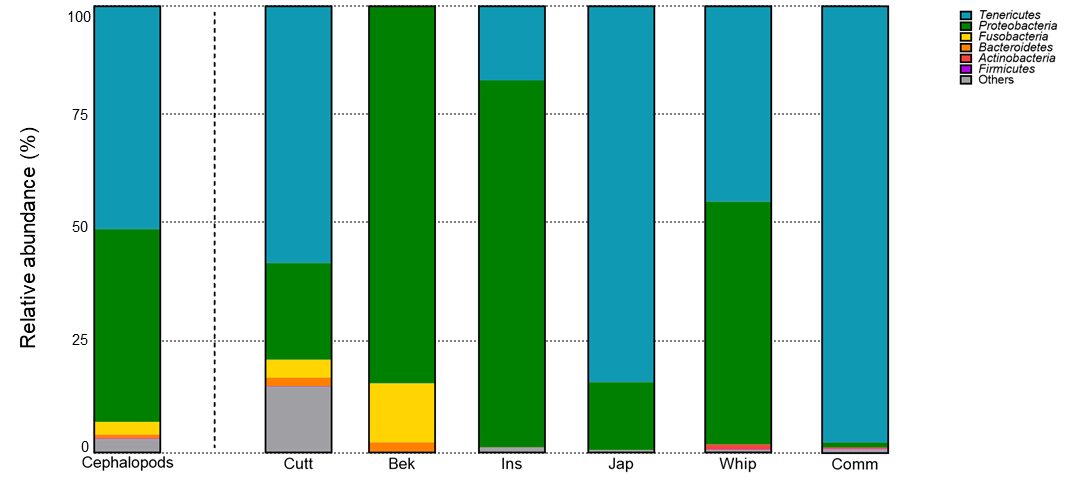


**Supplementary Fig. S3.** Gut microbial compositions of cephalopods. Bar charts of the relative abundance of bacterial phyla in six cephalopod species as well as the overall gut microbial composition of cephalopods. Only phyla with a relative abundance of > 1% are shown; those with an abundance of < 1% are classified as “Others.” Abbreviations: Cutt, cuttlefish; Bek, beka squid; Ins, Inshore squid; Jap, Japanese flying squid; Whip, whiparm squid; Comm, common octopus.


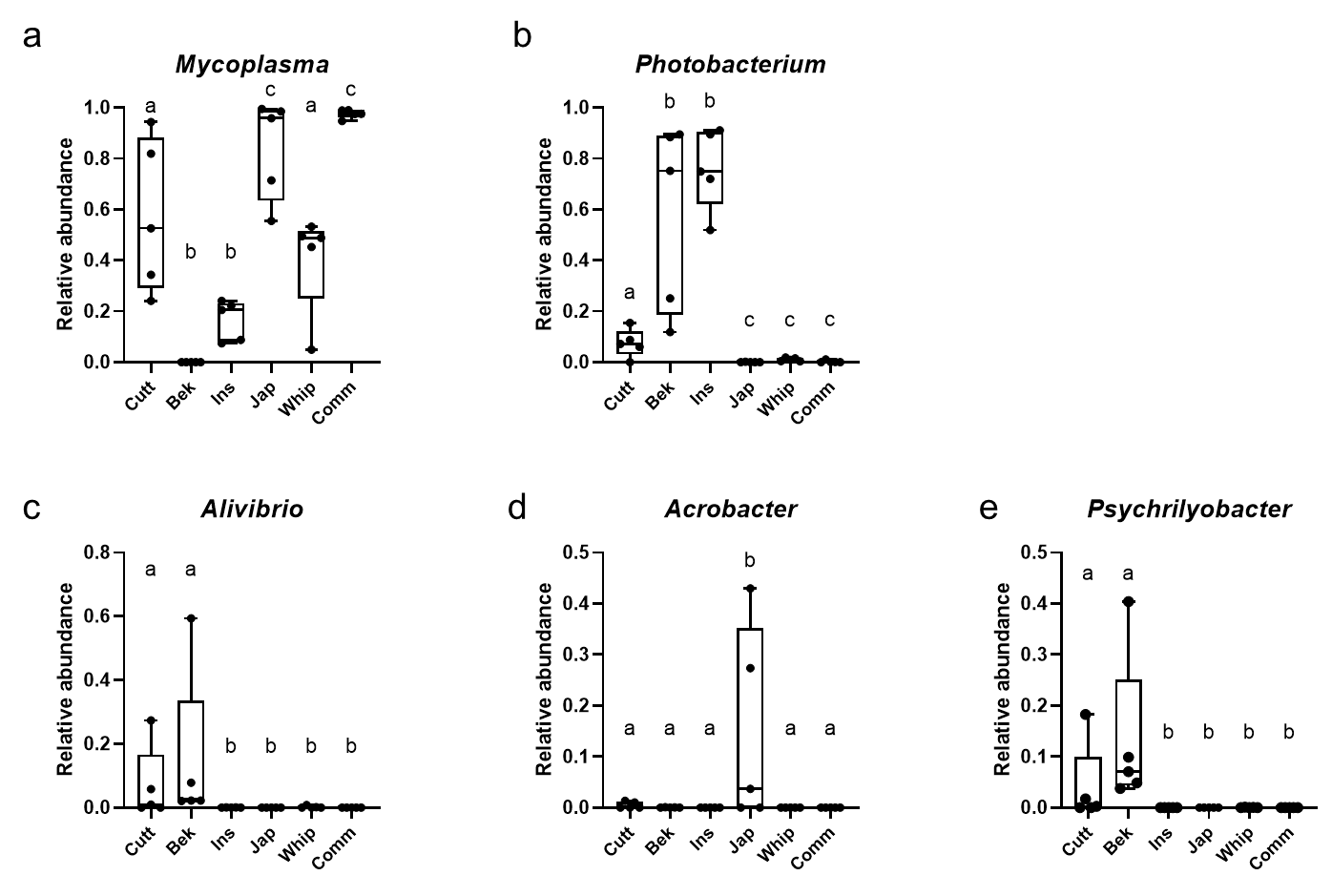


**Supplementary Fig. S4.** Distribution of core genera of cephalopods. Boxplot diagram of (a) *Mycoplasma,* (b) *Photobacterium,* (c) *Alivibrio,* (d) *Acrobacter,* and (e) *Psychrilyobacter*. The letters above the whisker indicate significant differences (p < 0.05) among groups (Mann-Whitney U test). Abbreviations: Cutt, cuttlefish; Bek, beka squid; Ins, Inshore squid; Jap, Japanese flying squid; Whip, whiparm squid; Comm, common octopus.


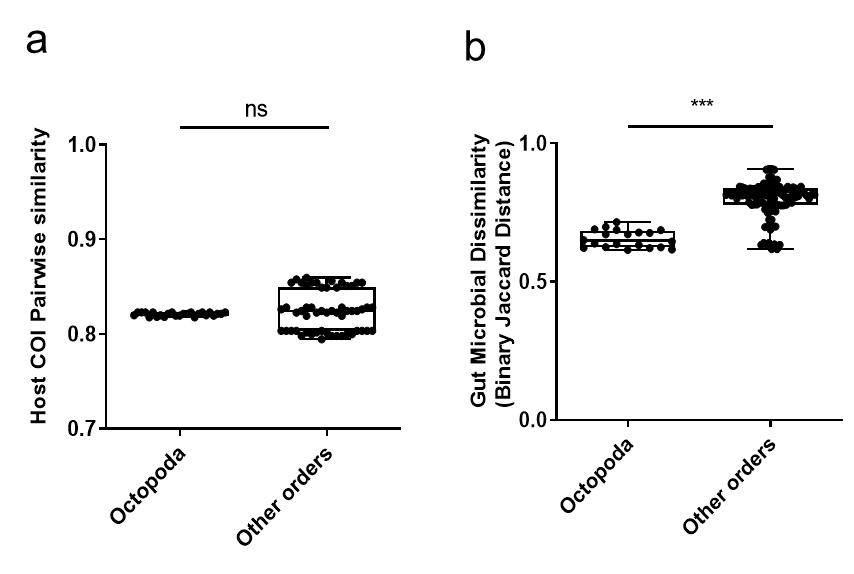


**Supplementary Fig. S5.** Comparisons of intra-order host COI similarity and microbial variation of *Octopoda* and other orders. Host similarity was calculated with pairwise COI sequence comparison. Microbial variation was calculated based on binary Jaccard distance. Asterisks indicate significant differences according to two-tailed Mann-Whitney U tests. *p < 0.05, **p < 0.01; ***p < 0.001.


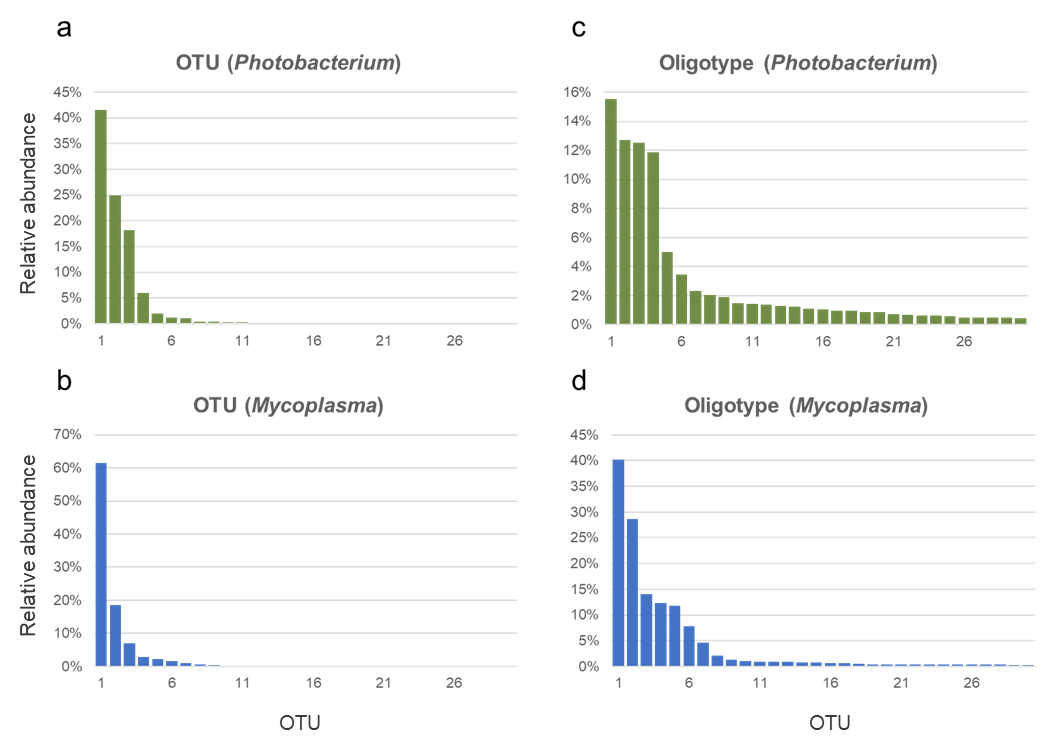


**Supplementary Fig. S6.** Distributions of *Photobacterium* and *Mycoplasma* OTUs and oligotypes in cephalopod gut microbiomes. The distributions of 97% clustered OTUs assigned to *Photobacterium* and *Mycoplasma* (a–b) are compared with re-clustered oligotypes and generated using the MED pipeline with aligned sequence reads that originally matched to *Photobacterium* and *Mycoplasma* by the QIIME 1.9.1 pipeline.


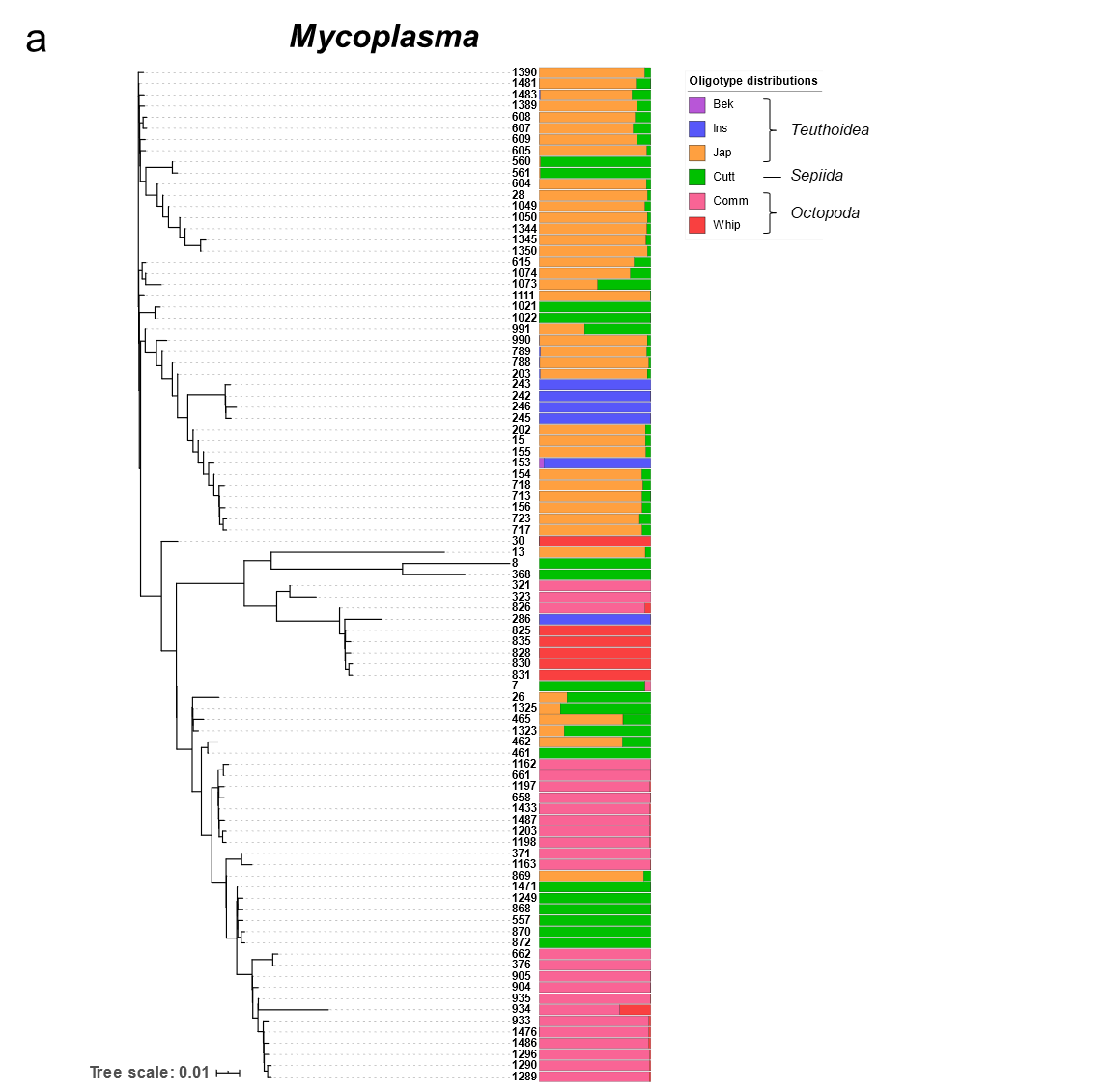


**
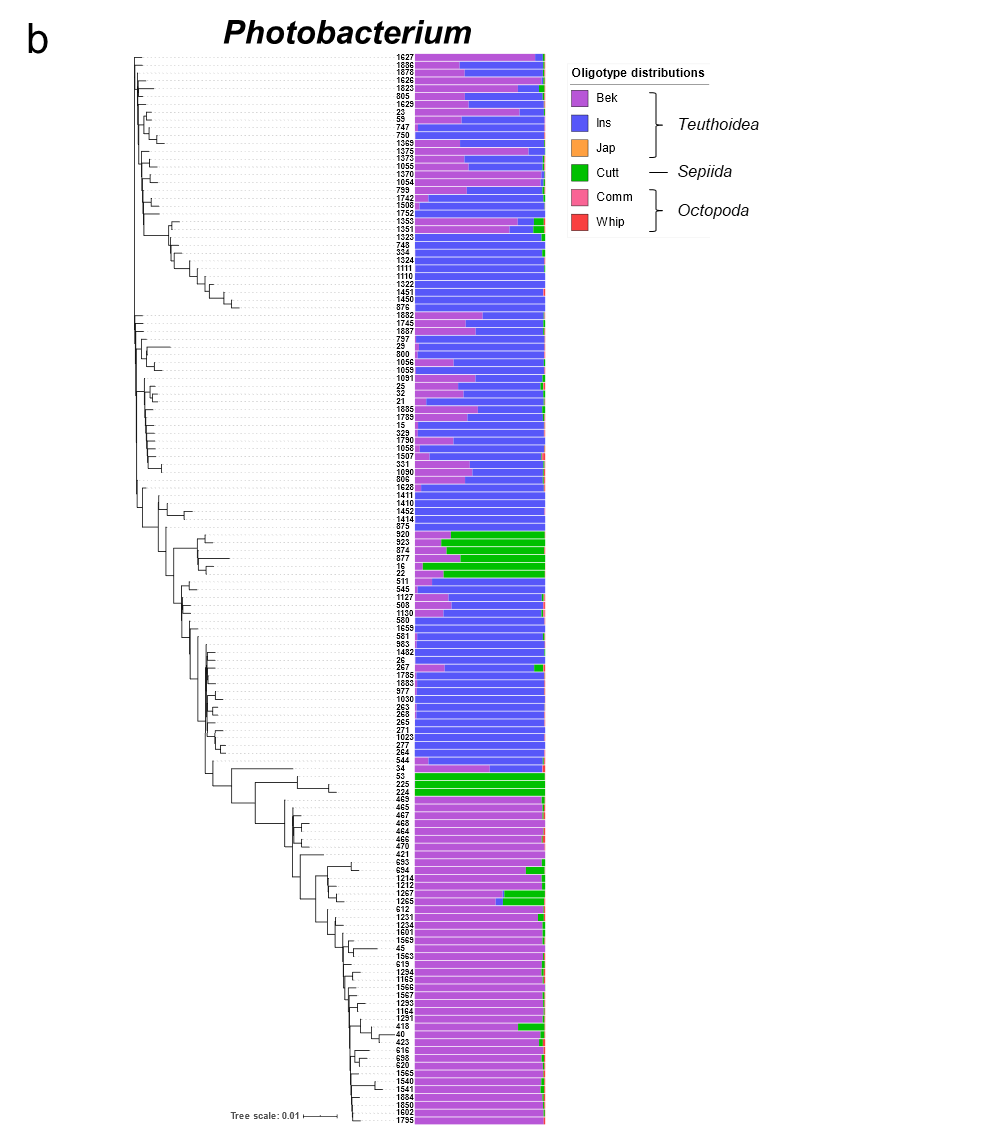
**

**Supplementary Fig. S7.** Phylogenetic trees of *Photobacterium* and *Mycoplasma* based on oligotypes (maximum likelihood tree with 1000 bootstrap replicates and the GTR + Gamma model). Bar graphs are color-coded to show the proportions of oligotypes assigned to *Photobacterium* (a) and *Mycoplasma* (b) in each cephalopod species. Abbreviations: Cutt, cuttlefish; Bek, beka squid; Ins, Inshore squid; Jap, Japanese flying squid; Whip, whiparm squid; Comm, common octopus.


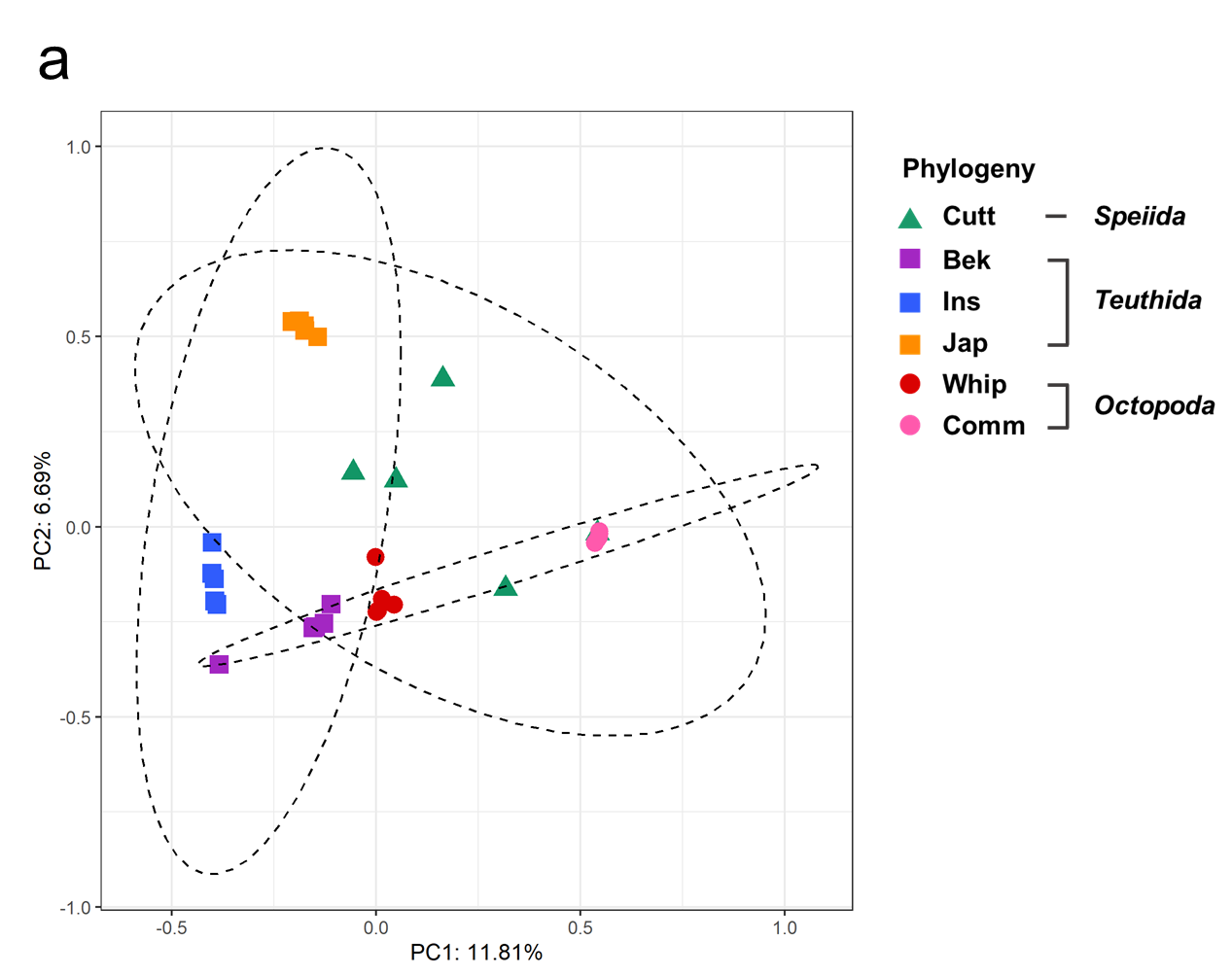


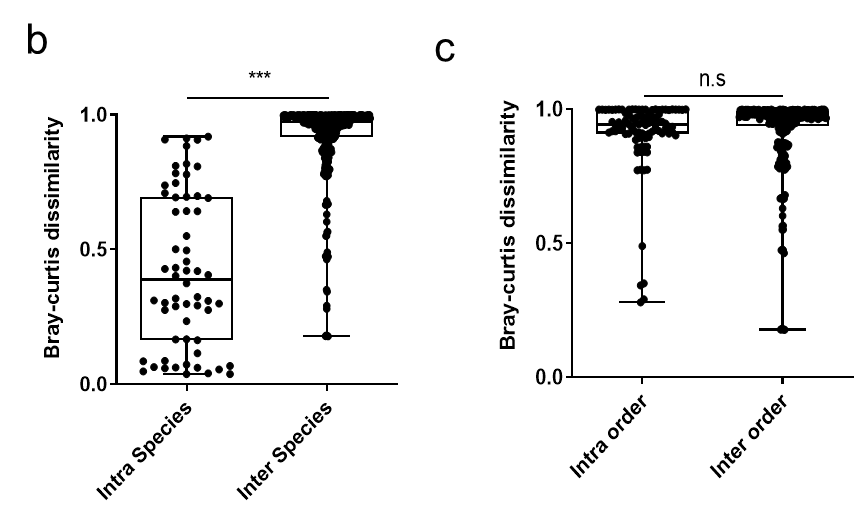


**Supplementary Fig. S8.** Beta-diversity analysis for cephalopod species using the Bray-Curtis index. Principal coordinates analysis (PCoA) of Bray-Curtis between cephalopod samples. The colors of the dots in the PCoA represent the host cephalopod species and their orders. (b–c) Comparisons of intra- and inter-specific (b) and intra- and inter-order (c) microbial variation based on the Bray-Curtis dissimilarity. Asterisks indicate significant differences according to two-tailed Mann-Whitney U tests. *p < 0.05, **p < 0.01; ***p < 0.001. Abbreviations: Cutt, cuttlefish; Bek, beka squid; Ins, Inshore squid; Jap, Japanese flying squid; Whip, whiparm squid; Comm, common octopus.

**
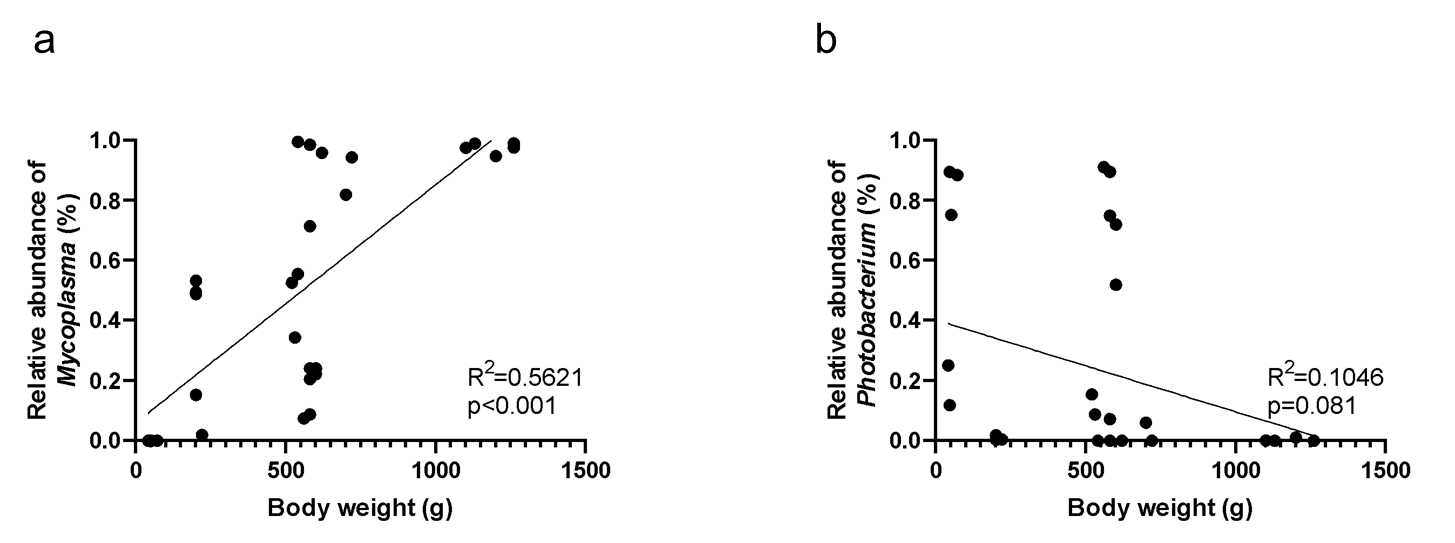
**

**Supplementary Fig. S9.** Linear regression analysis with the slope of the regression line. Correlation between body weight and relative abundance of *Mycoplasma* (a) was positive and significant, but body weight and relative abundance of *Photobacterium* (b) was negative but not significant.


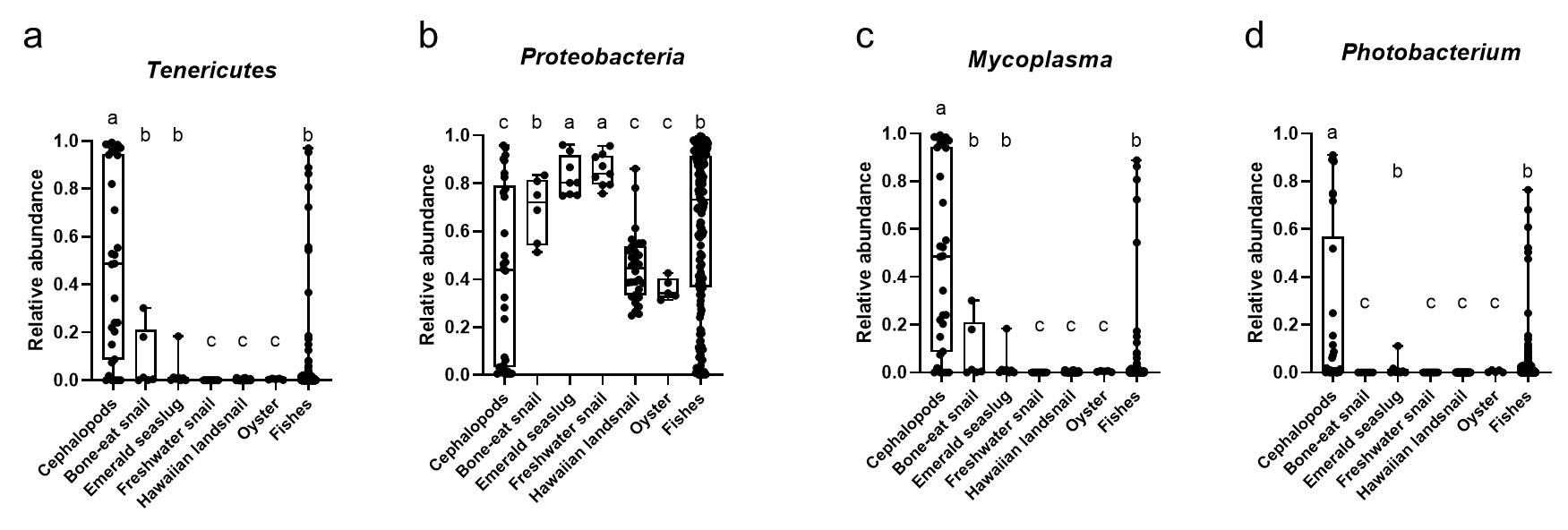


**Supplementary Fig. S10.** Boxplot diagram of the relative abundance for the phyla (a) *Tenericutes*, (b) Proteobacteria, and genera (c) *Mycoplasma*, and (d) *Photobacterium*. The letters above the whisker indicate significant differences (p < 0.05) among groups (Mann-Whitney U test).


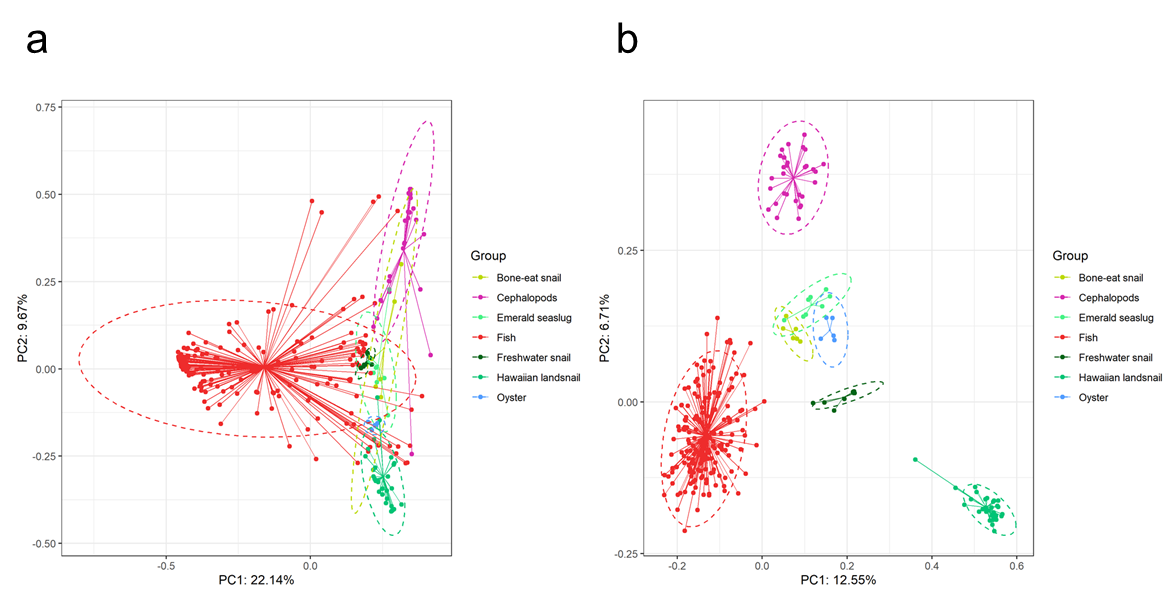


**Supplementary Fig. S11.** Beta-diversity analysis for mollusk and fish using the binary Jaccard and Bray-Curtis indices. PCoA plots with (a) binary Jaccard and (b) Bray-Curtis indices show different distribution patterns. The colors of the dots, centroids and ellipses in the PCoAs represent the group that each host belongs to
